# Defying the Odds: Small Cell Lung Cancer with Essential Thrombocythemia and Secondary Cancers

**DOI:** 10.7759/cureus.86093

**Published:** 2025-06-15

**Authors:** Mathew George, Thomas Mathew, Jonathon Vundum, Anita Mani, Israfil Baluwala

**Affiliations:** 1 Medcial Oncology, Calvary Mater, Newcastle, AUS; 2 University of Newcastle, School of Medicine and Public Health, Newcastle, AUS; 3 Medical Oncology, Calvary mater, Newcastle, AUS; 4 Pathology, Tamworth Rural Referral Hospital, New South Wales, AUS; 5 Hematology, Calvary Mater, Newcastle, AUS

**Keywords:** essential thrombocythemia, jak2 mutation, myeloproliferative neoplasm, secondary malignancies, small cell lung cancer

## Abstract

Small cell lung cancer (SCLC) is an aggressive malignancy with historically poor survival outcomes, especially in patients with significant comorbidities. This report presents a rare case of a 65-year-old woman with limited-stage SCLC and JAK2-positive essential thrombocythemia (ET), who subsequently developed multiple malignancies including papillary thyroid carcinoma, right-sided colon adenocarcinoma, and basal cell carcinoma. Despite serious complications such as subdural hematoma and intra-abdominal bleeding, the patient remains clinically stable over a decade after her initial diagnosis. Genetic testing revealed no hereditary cancer syndromes, suggesting a potential role of chronic inflammation and prior therapies in her cancer trajectory. This case underscores the importance of early aggressive therapy, vigilant long-term follow-up, and multidisciplinary coordination in managing complex oncologic and hematologic conditions.

## Introduction

Small cell lung cancer (SCLC) accounts for approximately 13-15% of all lung cancers and is characterized by rapid growth, early dissemination, and high relapse rates. Approximately 30% of SCLC cases present with limited-stage disease (LS-SCLC), for which standard treatment includes concurrent platinum-based chemotherapy and thoracic radiotherapy, often followed by prophylactic cranial irradiation (PCI) to mitigate brain metastasis risk [1,2]. While initial response rates are high, long-term survival remains uncommon.

Essential thrombocythemia (ET) is a chronic myeloproliferative neoplasm (MPN) defined by sustained thrombocytosis, JAK2 V617F mutation in most cases, and elevated risks of thrombosis, bleeding, and secondary malignancies [3]. Chronic inflammation is increasingly recognized as a driver of cancer in MPNs, potentially mediated by elevated cytokines (e.g., IL-6, TNF-α), oxidative stress, and immune dysregulation [4].

The coexistence of LS-SCLC, ET, and multiple secondary malignancies is exceedingly rare. This case demonstrates the feasibility of long-term survival under close multidisciplinary care and provides insight into the role of chronic inflammation in cancer development. Ongoing trials like ADRIATIC are investigating consolidation immunotherapy in LS-SCLC, potentially transforming future management [5].

## Case presentation

A 65-year-old woman with a 40 pack-year smoking history (ceased 10 years earlier) initially presented with transient ischemic symptoms including facial paresthesia, diplopia, and right arm weakness. Investigations revealed marked thrombocytosis (>950 × 10⁹/L), elevated hematocrit, and leukocytosis. Bone marrow biopsy confirmed JAK2 V617F-positive ET. She commenced aspirin, later switched to pegylated interferon-alpha (Pegasys) after developing hepatotoxicity from hydroxyurea.

**Figure 1:**
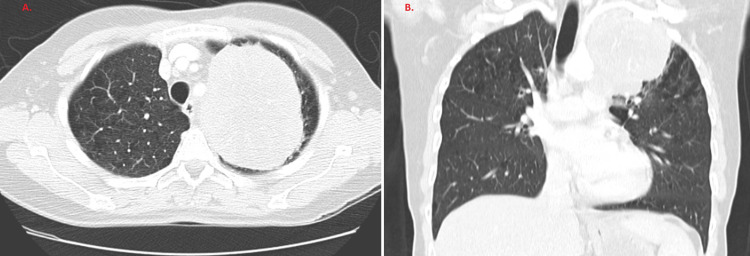
CT Chest. September 2010 demonstrating 10.6 cm mass in left upper hemithorax. A. Axial view. B. Coronal view.

**Figure 2:**
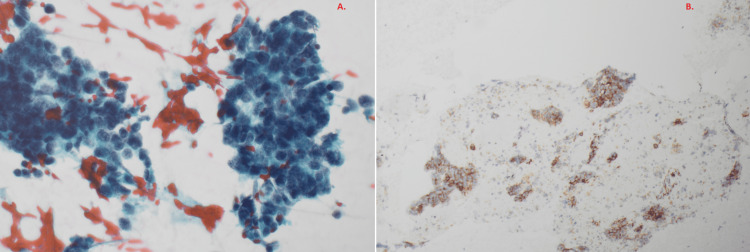
Histopathology results demonstrating small cell carcinoma of lung. A. Papanicolaou (Pap) stain. B. CD56 stain.

In September 2010, she developed persistent cough, hemoptysis, and left-sided chest pain. CT imaging revealed a 10.6 cm mass in the left upper lobe with mediastinal lymphadenopathy (Figure 1). Fine needle aspiration demonstrated malignant small round cells with nuclear molding. Immunohistochemistry was positive for CD56 and thyroid transcription factor-1 (TTF-1), supporting a diagnosis of LS-SCLC (Figure 2)..

She received four cycles of concurrent carboplatin and etoposide chemotherapy with thoracic radiotherapy. Prophylactic cranial irradiation was administered post-treatment due to the high risk of brain metastases.

Post-treatment surveillance (2012-2014) showed no evidence of recurrence. PET scans (2015-2016) demonstrated stable post-radiotherapy changes. Platelet counts remained elevated (>1000 × 10⁹/L), and Pegasys was continued.

Subsequently, the patient developed several additional malignancies. A PET-detected thyroid nodule was confirmed as papillary thyroid carcinoma; she underwent total thyroidectomy followed by radioiodine treatment. Later, iron deficiency prompted colonoscopy, revealing right-sided colon adenocarcinoma. She had a right hemicolectomy without chemotherapy. Complications included a subdural hematoma and intra-abdominal bleeding, likely exacerbated by ET-associated platelet dysfunction.

Other diagnoses included basal cell carcinoma of the nose (treated with radiotherapy) and a left frontal meningioma, now pending surgical resection. Genetic testing revealed no mutations in common cancer predisposition genes (*APC*, *AXIN2*, *BMPR1A*, *EPCAM*, *MLH1*, *MSH2*, *MSH6*, *MUTYH*, *PTEN*).

Over 14 years post-SCLC diagnosis, the patient remains clinically stable. Surveillance imaging confirms no recurrence. Platelet counts are controlled with weekly Pegasys (45-90 mcg). The meningioma was slowly enlarging.

## Discussion

This case demonstrates prolonged survival in a patient with LS-SCLC and JAK2-mutated ET who developed multiple metachronous malignancies. Such extended survival is exceptional in LS-SCLC, where median overall survival is 15-20 months and five-year survival rates hover between 20-30% in select populations [1,2]. Early diagnosis, aggressive multimodal treatment, prophylactic cranial irradiation, and comprehensive follow-up likely contributed to this favorable outcome.

The proinflammatory state characteristic of ET, particularly in JAK2 V617F-positive cases, is increasingly associated with elevated cancer risk [4,6]. Chronic interferon therapy, while cytoreductive, may exacerbate systemic inflammation, alter immune surveillance, and promote carcinogenesis [5,7]. Although this patient’s inflammatory markers were not monitored longitudinally, the development of multiple primary malignancies during prolonged Pegasys therapy supports this association.

Despite the intrinsic hemorrhagic and thrombotic risks of ET, the patient successfully underwent several major interventions. This underscores the importance of personalized, multidisciplinary management. Previous reports described similar patterns of malignancy in MPNs, and population-based cohort data support this association [8].

## Conclusions

This case highlights the potential for long-term survival in a patient with LS-SCLC and ET who developed multiple subsequent malignancies. It underscores the importance of early, aggressive treatment, ongoing surveillance, and coordinated multidisciplinary care. The patient's outcome supports further investigation into the role of chronic inflammation, MPNs, and immune dysregulation in cancer pathogenesis and survivorship.
